# Aberrant Expression of TIMP-2 and PBEF Genes in the Placentae of Cloned Mice Due to Epigenetic Reprogramming Error

**DOI:** 10.1371/journal.pone.0166241

**Published:** 2016-11-17

**Authors:** Hong Rye Kim, Jae Eun Lee, Reza Kheirkhahi Oqani, So Yeon Kim, Teruhiko Wakayama, Chong Li, Su Jin Sa, Je Seok Woo, Dong Il Jin

**Affiliations:** 1 Department of Animal Science & Biotechnology, Chungnam National University, Daejeon, Republic of Korea; 2 Faculty of Life and Environmental Science, University of Yamanashi, Yamanashi, Japan; 3 School of Medicine, Tongi University, Shanghai, China; 4 Department of Animal Resource Development, National Institute of Animal Science, Cheonan, Republic of Korea; University of Texas at Austin Dell Medical School, UNITED STATES

## Abstract

Cloned mice derived from somatic or ES cells show placental overgrowth (placentomegaly) at term. We had previously analyzed cloned and normal mouse placentae by using two-dimensional gel electrophoresis and mass spectrometry to identify differential protein expression patterns. Cloned placentae showed upregulation of tissue inhibitor of metalloproteinase-2 (TIMP-2), which is involved in extracellular matrix degradation and tissue remodeling, and downregulation of pre-B cell colony enhancing factor 1 (PBEF), which inhibits apoptosis and induces spontaneous labor. Here, we used Western blotting to further analyze the protein expression levels of TIMP-2 and PBEF in cloned placentae derived from cumulus cells, TSA-treated cumulus cells, intracytoplasmic sperm injection (ICSI), and natural mating (NM control). Cloned and TSA-treated cloned placentae had higher expression levels of TIMP-2 compared with NM control and ICSI-derived placentae, and there was a positive association between TIMP-2 expression and the placental weight of cloned mouse concepti. Conversely, PBEF protein expression was significantly lower in cloned and ICSI placentae compared to NM controls. To examine whether the observed differences were due to abnormal gene expression caused by faulty epigenetic reprogramming in clones, we investigated DNA methylation and histone modification in the promoter regions of the genes encoding TIMP-2 and PBEF. Sodium bisulfite sequencing did not reveal any difference in DNA methylation between cloned and NM control placentae. However, ChIP assays revealed that the level of H3-K9/K14 acetylation at the *TIMP-2* locus was higher in cloned placentae than in NM controls, whereas acetylation of the *PBEF* promoter was lower in cloned and ICSI placenta versus NM controls. These results suggest that cloned placentae appear to suffer from failure of histone modification-based reprogramming in these (and potentially other) developmentally important genes, leading to aberrant expression of their protein products. These changes are likely to be involved in generating the abnormalities seen in cloned mouse placentae, including enlargement and/or a lack of proper placental function.

## Introduction

The placenta connects the developing fetus to the mother’s body, and its growth is an essential characteristic of mammalian pregnancy. Successful placental development is important for the survival of the growing conceptus, as the placenta specifically nurtures the fetus, protects it from harmful waste, executes the exchange of respiratory gases and a multitude of nutrients, and acts as an immunological barrier [1]. Therefore, the study of placentation is considered key to understanding the pregnancy failures of cloned animals, the pathogenesis of some congenital diseases, and the transplacental transmission of teratogenic microbial agents [2].

Many of the fetal losses and phenotypic abnormalities observed among cloned mammals are associated with placental abnormalities [2,3,4]. For example, cloned mouse fetuses have been shown to develop to term and yield placental overgrowth (placentomegaly) regardless of the sex or source of the donor nuclei [5,6,7,8]. This placentomegaly has been associated with expanded spongiotrophoblast layers, increased glycogen cell numbers, and enlarged trophoblastic cells [9]. Trophoblast cells develop into some extra-embryonic membranes and much of the placenta [10], so perfect reprogramming of transferred somatic nuclei (which is needed to properly establish the trophoblast cell lineage) is required for the correct development of reconstructed embryos. It has been speculated that placentomegaly in cloned mice could reflect the acquisition of epigenetic abnormalities, at least partly via inadequate nuclear reprogramming. Consistent with this hypothesis, errors in the epigenetic reprogramming of the somatic cell genome have been associated with the expressional dysregulation of developmentally imprinted genes in cloned embryos, fetuses and placentae, and abnormalities in the resulting cloned animals [11,12,13,14,15]. In this context, numerous researchers have observed abnormal gene expression patterns following somatic cell nuclear transfer [16,17,18,19]. However, the existence, importance, and regulation of epigenetic modification-related abnormalities in gene expression have not yet been fully elucidated in the context of placentation.

Chromatin-modification-related changes in gene expression [20], called “epigenetic changes,” support: various processes that require accurate gene activation/inactivation during development; the assembly of histones and histone variants into nucleosomes; and the remodeling of chromatin-associated proteins (e.g., linker histones, polycomb proteins, nuclear scaffold proteins, and transcription factors) [21]. Changes in chromatin configuration are critical to normal development, and are primarily determined by genomic DNA methylation and the histone acetylation/methylation status. Successful placental development depends on precisely regulated gene expression and may be negatively influenced by the abnormal expression of developmentally significant genes [22]: aberrant gene expression in the placenta (i.e., that arising via epigenetic error) may alter the placenta and perhaps even the conceptus.

We had previously used two-dimensional gel electrophoresis and mass spectrometry to conduct a global proteomic analysis of cloned and normal mouse placentae, in an effort to identify differential protein expression patterns [17,18,19]. TIMP-2 and PBEF which was related with pregnancy and placenta development, are abnormally expressed in cloned bovine and mouse placentae [17,18,19] and differentially expressed during pregnancy [23, 24]. TIMP-2 has been associated with trophoblastic invasion and extracellular matrix remodeling during pregnancy [25], while PBEF appears to function at the proximal end of the labor initiation pathway [26,27,28]. The development of mouse cloned embryos could be improved by trichostatin A (TSA, histone deacetylase inhibitor) treatment which could adjust pre-existing epigenetic state of donor cells or reconstructed embryos [29]. To examine the potential contribution of epigenetic modification to this gene expression process, we herein analyzed the protein and mRNA levels of TIMP-2 and PBEF in cloned, TSA-treated cloned, intracytoplasmic sperm injection (ICSI)-derived and normal mating-derived (NM control) placentae. Following their confirmation as differentially expressed proteins, we examined the DNA methylation and histone acetylation patterns in their gene promoter regions to determine whether their expression changes were associated with differences in the epigenetic programming of cloned versus normal mouse placentae. The novel identification of these genes as being differentially expressed in the placentae of cloned mice and our assessment of their epigenetic regulation provide important new insights into the molecular mechanisms underlying placental development. In the future, this may help researchers improve the efficiency of cloning.

## Materials and Methods

### Placental samples

All animal procedures were approved by the Institutional Animal Care and Use Committee of Chungnam National University. Cloned, TSA-treated, normal mating-derived (NM controls), and ICSI-derived mouse placentae were generated as previously described [27]. The mice were maintained on a 14L:10D schedule and allowed free access to food and water. To generate NM controls, 6- to 8-wk-old B6D2F1 females were housed with adult B6D2F1 male mice and examined daily for vaginal plugs. Noon of the day on which a vaginal plug was found was designated as 0.5 days postcoitum (dpc). Cesarean sections were performed at 18.5 dpc, and the wet weights of fetuses and placentae were recorded separately. To generate ICSI-derived placentae, B6D2F1 mice were used as the sources of oocytes and spermatozoa, and embryos were generated and transferred to recipient surrogate mothers (ICR mice) as previously described [28]. To generate cloned placentae, the nuclei of B6D129F1 embryonic stem (ES) cells were injected into enucleated B6D2F1 oocytes; the reconstituted embryos were incubated for 72 h; and those that developed to the morula or blastocyst stages were transferred to the uteri of 2.5 dpc pseudopregnant ICR females, as previously described [5]. TSA-treated cloned placentae were generated using standard mouse cloning procedures [29]. Briefly, donor nuclei from somatic cells were injected into enucleated oocytes, and the reconstructed oocytes were activated by culture in Ca^2+^-free CZB medium containing 5 mM Sr^2+^. After 6 h, the activation medium was changed to KSOM. For TSA treatments, the activated oocytes were cultured in KSOM including TSA for 14 h, and then transferred to KSOM without TSA. The resulting cloned embryos were subjected to embryo transfer into surrogate mothers on the second day. Concepti were collected by Cesarean section at 18.5 dpc, and four placental samples per ICSI, cloned, TSA treatment and natural mating separated and frozen until use. Anesthesia was induced with ketamine (100 mg/kg) and xylazine (16 mg/kg), and 100% CO_2_ gas was used for sacrifice in this experiment.

### Protein extraction and Western blot analysis

Each placental sample was mixed with an equal volume of lysis buffer A containing 1% SDS, 1 mM PMSF, protease inhibitor (Roche Diagnostics), and 100 mM Tris-HCl. The samples were sonicated, and the resulting protein lysates were treated with 100 U/mL endonuclease (Sigma) and quantified using a Bradford assay kit (Bio-Rad). Total proteins (30 μg) were resolved by 12% SDS-PAGE and electro-transferred to PVDF membranes (Bio-Rad). Mouse anti-TIMP-2 (diluted 1:1000; Abcam), rabbit anti-β-actin (diluted 1:5000; Abcam), and rabbit anti-PBEF (diluted 1:10,000; Bethyl) antibodies were used to measure the expression level of each protein.

### RT- PCR

Total RNA was extracted from placentae using the AccuZol^™^ reagent (Bioneer). Quantitative real-time RT-PCR using fluorogenic 5’nuclease assay technology with TaqMan^®^ probes and primers (Applied Biosystems) were employed for RNA expression analysis. Amplification of individual genes was performed on the Applied Biosystems 7300 Real-Time PCR System using TaqMan^®^ Universal PCR Master Mix and a standard thermal cycler protocol. TaqMan^®^ Gene Expression Assay Reagents for mouse TIMP-2, mouse PBEF, and mouse GAPDH were used for specific probes and primers of PCR amplifications. The threshold cycle (C_T_) was determined and relative quantification was calculated by the comparative C_T_ method as described previously. [30] The experiments were conducted in triplicate, and the results were normalized with respect to the mRNA expression level of GAPDH.

### Isolation and bisulfite treatment of genomic DNA

Genomic DNA was isolated from cloned, TSA-treated cloned, ICSI-derived, and NM control placentae using phenol/chloroform extraction and ethanol precipitation. The purified genomic DNA (1 μg) was subjected to sodium bisulfate treatment using an MSP kit (In2Gen). The treated DNA was purified according to the protocol provided with the MSP kit, precipitated with ethanol, and resuspended in 20 μl of distilled water. The *TIMP-2* and *PBEF* promoter regions were PCR amplified using primers designed to convert the cytosines to uracils, as follows: for *TIMP-2*, MF1(F) 5'-TTTTTTAGGGATAAGGTTTGAGTTTTAT-3' and MR1(R) 5'-TTATACCCAACCTAACACAAACTAC-3', and MF2(F) 5'-GTAGTTTGTGTAGGTTGGGTTT-3' and MR2(R) 5'-CAAACTTTATATCCTCTTTATCAAAA-3'; and for *PBEF*, PM3(F) 5'-AGTGGGTGGTTTTTTGGTTTTA-3' and PM3(R) 5'-CCCATCCCRAAAACAAAA-3', and PM4(F) 5'-GGGYGTAGTTGAGGTAGAG-3' and PM4(R) 5'-ACAAAAAAAAAAATAAACTTCRC-3'. Amplification was performed using an ExTaq Hot Start kit (Takara). The resulting PCR products were ligated into T-plasmids using the pGEMP^®^P-T Easy Vector System (Promega), and 10 subclones per experiment were randomly picked and sequenced.

### Chromatin immunoprecipitation assays

Chromatin immunoprecipitation (ChIP) was performed as previously described [31] using antibodies against acetylated histone H3-K9/K14 (acetyl-lysines 9 and 14) and H4-K5 (acetyl-lysine 5), as both of these modifications have been correlated with gene activation [31]. The anti-acetyl-histone H3-K9/K14 and anti-acetyl-H4-K5 antibodies were obtained from Upstate. Tissues samples (20~40 mg/ml) were frozen, homogenized, and then centrifuged. The pellets were resuspended in 1 ml of 4% formaldehyde in PBS, crosslinked at 37°C for 30 min, and then centrifuged at 2000 g for 5 min at 4°C. The resulting pellets were resuspended in SDS lysis buffer (150 mM NaCl, 25 mM Tris∙HCl, pH 7.5, 5 mM EDTA, 1% Triton X-100, 0.1% SDS, and 0.5% sodium deoxycholate; Upstate) per the manufacturer’s instructions. The samples were sonicated four times for 10 sec each with the sonicator (Hielscher) set at 13% maximal power to generate fragments of < 500 bp in length, as confirmed by agarose gel electrophoresis. For ChIP, the sonicated chromatin (150 μl) was diluted 10-fold, cleared with salmon sperm DNA/protein A-agarose (80 μl), and then purified with specific antiserum (2–5 μl) and protein A-agarose (60 μl). The crosslinking was then reversed, the samples were treated with proteinase K, and the chromatin-bound DNA was precipitated and diluted in 100 μl of low-TE buffer (1 mM Tris, 0.1 mM EDTA). Real-time PCR amplification was performed in triplicate using SYBR Green (Roche Diagnostics) on a Rotor Gene 2000 PCR machine (Cobett Research), with the following primers: for *TIMP-2*, TIMP-2(F) 5'-TGTGTGGCTGCTTAGATTGC-3' and TIMP-2(R) 5'-CAGTCTCACCTGCTGAGTGC-3'; and for *PBEF*, PBEF(F) 5’-CTTTCCCTAAGACGCAAAGG-3' and PBEF(R) 5'-ACGATGGATGGAATCTTTGG-3'. The fold induction over the input was calculated using the 2^-ΔΔCT^ method [30].

### Statistical analysis

Significant differences among samples were determined by ANOVA followed by Duncan’s multiple range tests, using the GLM found in the SAS package (SAS Institute). P-values < 0.05 were considered statistically significant.

## Results

### Overgrowth of cloned placentae

**Fig 1:**
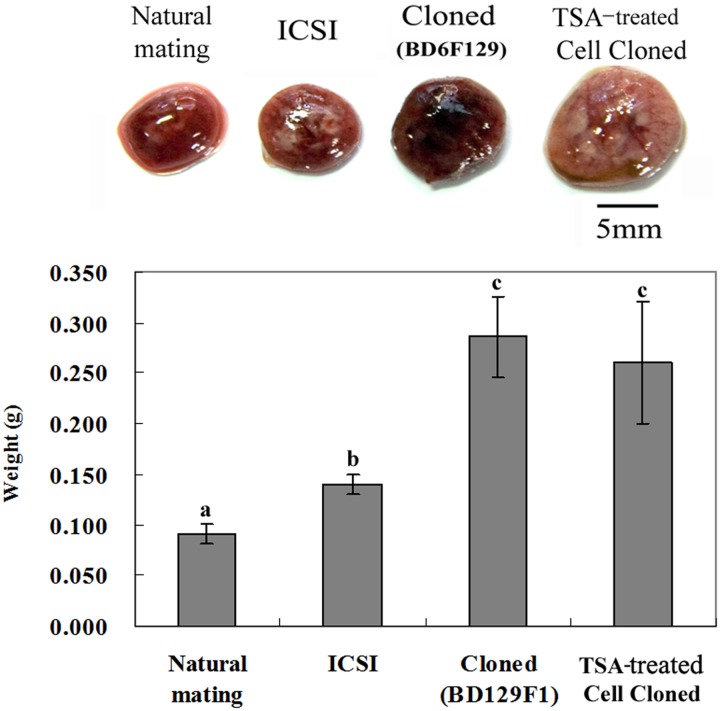
Weights of cloned mouse placentae measured immediately after Cesarean section. Size differences between NM control, ICSI-derived, cloned, and TSA-treated cloned placentae are shown. Four placental samples per ICSI, cloned, TSA treatment and natural mating were collected. Error bars show the standard error (SE). Different letters (a-c) above the bars denote statistically significant differences (p < 0.05).

The weights and sizes of mouse cloned and TSA-treated cloned placentae were compared with those of ICSI-derived and NM control placentae. As shown in Fig 1, the cloned and TSA-treated cloned placentae tended to have larger diameters than the NM control placentae (Fig 1), and weighed (on average) 310% (cloned) and 282% (TSA-treated cloned) of the mean weights of the NM control (0.092 g). There was no significant difference in weight between the cloned and TSA-treated cloned placentae (average 0.286 and 0.260 g, respectively). In addition, ICSI-derived placentae (0.140 g) tended to have higher weights (152%) than the NM control (0.092 g).

### Abnormal protein expression of TIMP-2 and PBEF in cloned placentae

**Fig 2:**
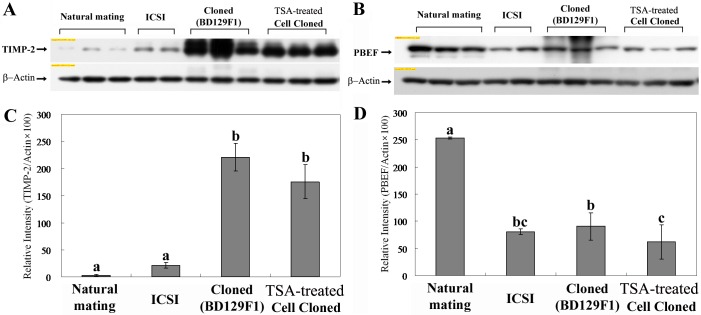
Western blot analyses of TIMP-2 and PBEF expression levels in in NM control, ICSI-derived, cloned, and TSA-treated cloned placentae. (A) Representative bands detected with anti-TIMP-2 and anti-β-actin antibodies. (B) Representative bands detected by anti-PBEF and anti-β-actin antibodies. (C, D) Protein levels were subjected to densitometric quantification followed by normalization against the protein level of β-actin. Four replicates were performed. The mean of expression levels with different superscripts (a-c) differ significantly (P < 0.05). Error bars show the standard error (SE).

We previously reported two proteins that appear to be differentially expressed during pregnancy and placental development: TIMP-2 and PBEF. The former contributes to extracellular matrix degradation and remodeling and is upregulated in cloned placentae [17,18,19], while the latter contributes to inhibiting apoptosis and inducing spontaneous labor [26] and is down-regulated in cloned placentae [19]. Here, we used Western blotting to analyze the protein expression patterns of TIMP-2 and PBEF in cloned, TSA-treated cloned, ICSI-derived and NM control placentae. A TIMP-2-immunoreactive band of the appropriate size (24 kDa) was found in all tested samples (Fig 2A). Consistent with our previous results, cloned and TSA-treated cloned placentae expressed markedly higher levels of TIMP-2 protein than ICSI-derived and NM control placentae (Fig 2A and 2C). PBEF, which was successfully detected as a ~56-kDa band in all tested samples (Fig 2B), was observed at significantly lower levels in cloned, TSA-treated cloned and ICSI-derived placentae compared to NM controls (Fig 2B and 2D). Notably, ICSI-derived and TSA-treated cloned mouse placentae had comparable expression levels of PBEF (Fig 2D).

### Abnormal mRNA expression of TIMP-2 and PBEF in cloned placentae

**Fig 3:**
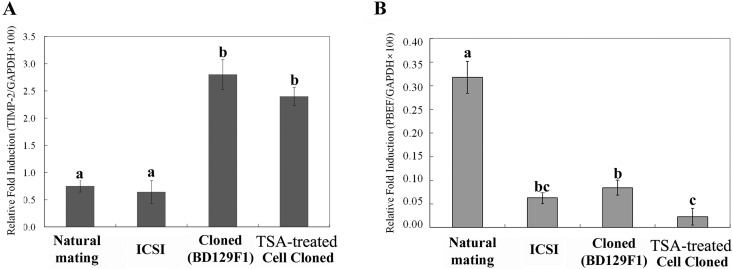
The mRNA expression levels of *TIMP-2* and *PBEF* in NM control, ICSI-derived, cloned, and TSA-treated cloned placentae. (A) *TIMP-2* mRNA level (relative fold induction) in RT-PCR. (F) *PBEF* mRNA level (relative fold induction) in RT-PCR. Four replicates were performed. The mean of expression levels with different superscripts (a-c) differ significantly (P < 0.05). Error bars show the standard error (SE).

Real time**-**PCR (RT-PCR) was used to examine the mRNA expression patterns of *TIMP-2* and *PBEF* in all tested samples. The cloned and TSA-treated cloned placentae showed higher *TIMP-2* expression levels compared to the NM control and ICSI-derived placentae (Fig 3A). The mRNA expression levels of *PBEF* were significantly lower in cloned, TSA-treated cloned, and ICSI-derived placentae compared with NM control placentae (Fig 3B). These findings are consistent with the observed protein expression pattern of PBEF (Fig 2B and 2D).

### Abnormal epigenetic modification of the *TIMP-2* and *PBEF* promoter regions

**Fig 4:**
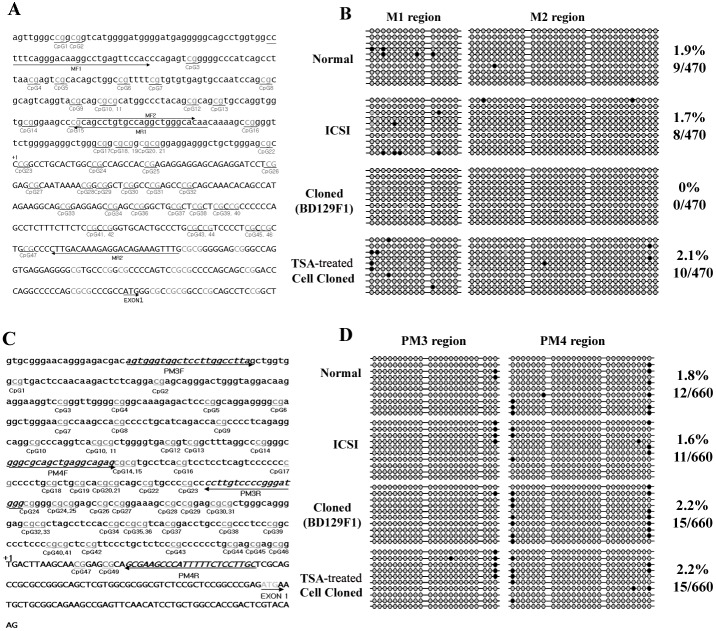
Methylation patterns at the promoter regions of *TIMP-2* and *PBEF*. (A) Positions of the 47 CpG dinucleotides within the 641-bp region upstream of the *TIMP-2* transcription initiation start site. (B) The methylation status of these CpG sites in NM control, ICSI-derived, cloned, and TSA-treated cloned placentae, as determined by bisulfite sequencing of at least 20 independent PCR clones. (C) The 49 CpG dinucleotides within the 652-bp region upstream of *PBEF*. (D) The methylation status of the CpG sites shown in (C). Gray circle, unmethylated; black circle, methylated. Percentages indicate the proportions of total available CpG sites that are methylated.

**Fig 5:**
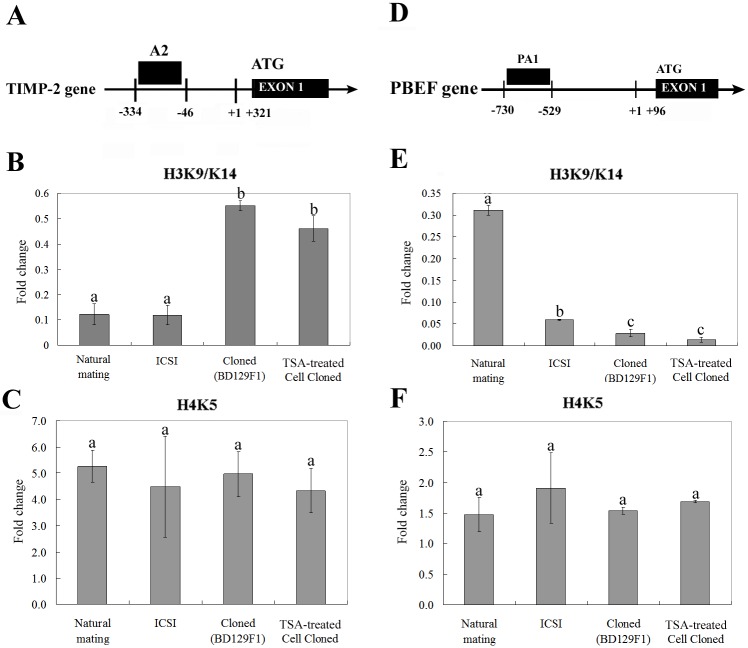
Histone modifications at the *TIMP-2* and *PBEF* promoter regions. (A) Schematic of the *TIMP-2* promoter region. Abbreviations: +1, the transcription start site; and A2, site analyzed for histone acetylation. (B) H3-K9/K14 acetylation of *TIMP-2* in NM control, ICSI-derived, cloned, and TSA-treated cloned placentae. (C) Graph showing the H4-K5 acetylation status of *TIMP-2*. (D) Schematic of the *PBEF* promoter region. Abbreviations: +1, the transcription start site; and PA1, site analyzed for histone acetylation. (E) H3-K9/K14 acetylation of *PBEF* was assessed by real-time PCR. (F) Graph showing the H4-K5 acetylation status of *PBEF*. The minimum value was standardized to 1. Four replicates were performed. The mean of fold change with different superscripts (a-c) differ significantly (P < 0.05). Error bars show the standard error (SE).

To evaluate whether the observed expression changes in TIMP-2 and/or PBEF were associated with epigenetic changes, we examined the DNA methylation and acetylation levels of their gene promoter regions. Bisulfite sequencing was used to monitor the DNA methylation levels in the *TIMP-2* promoter region, which encompassed 641 bp and included 47 CpG sites (Fig 4A). As shown in Fig 4B, most of the CpG sites in the *TIMP-2* promoter were unmethylated, and there was no significant difference in methylation status between normal and cloned placentae. Similar results were obtained with the *PBEF* promoter, which did not demonstrate any difference in methylation status (Fig 4C and 4D). Next, ChIP assays were used to examine the acetylation levels of the *TIMP-2* and *PBEF* genes in cloned, TSA-treated cloned, ICSI, and NM control placentae (Fig 5). Chromatin fragments were precipitated using antibodies against acetylated histone H3 (acetyl-lysines 9 and 14) and H4 (acetyl-lysines 5), and real-time PCR was used to amplify fragments encoding the promoter regions of *TIMP-2* and *PBEF*. Our results revealed that the H3K9 and -K14 acetylation levels of the *TIMP-2* gene in ICSI and NM control mouse placentae were significantly lower than those in cloned and TSA-treated cloned placentae (Fig 5B). This pattern was similar to that observed for the protein expression of TIMP-2 (Fig 2A and 2C). With respect to the *PBEF* gene promoter, the H3K9 and -K14 acetylation levels in cloned, TSA-treated, and ICSI-derived placentae were considerably lower than those in NM control placentae and those of the ICSI mouse placentae were comparable to those of TSA-treated cloned placenta (Fig 5E). This was consistent with the patterns observed for PBEF protein and mRNA expression (Figs 2B, 2D, 3B, 3D and 3F). In contrast, no significant difference in H4K5 acetylation was observed for the *TIMP-2* or *PBEF* promoter regions in any tested sample (Fig 5C and 5F).

## Discussion

Fetal growth and development during pregnancy is supported by the placenta, which is a highly specialized organ that precisely ensures the efficient exchange of nutrients and waste products between the maternal and fetal circulatory systems. During the gestation of cloned mice and bovines, researchers have observed a high frequency of hypertrophic placentae [3, 11]. In mice, a comparative study of placental weight at term showed that placentae derived from somatic cell nuclear transfer tended to show placental overgrowth [11]. Placentomegaly in cloned mouse concepti, which is caused by expansion of the spongiotrophoblast layer, has been speculated to reflect abnormal gene expression [11]. We previously identified several placental proteins that were up- and downregulated in cloned animal placentae and could be used to distinguish between cloned and normal placentae [19]. Here, we quantitatively determined the mRNA and protein expression patterns for two of them, TIMP-2 and PBEF, in the placentae of cloned, TSA-treated cloned, ICSI-derived and NM control mice.

TIMP-2 has been associated with extracellular matrix remodeling [32,33], and we previously reported that it is overexpressed in cloned placentae, particularly at the end of gestation [17,18,19]. High-level TIMP-2 secretion from binucleate giant cells in bovine placentae reportedly inhibits the proteolytic activity of MMP-2 (matrix metalloproteinase-2), leading to decreased extracellular matrix degradation during the prepartum period [34]. At the end of pregnancy, the number of binucleate giant cells decreases, as does TIMP-2 protein production [34]. In addition, enzymatic extracellular matrix degradation increases, the placenta detaches, and the fetal membranes are released [33]. Thus, proper control of TIMP-2 secretion may be important for successful placentation and parturition. In the present study, markedly higher levels of TIMP-2 expression were observed in cloned and TSA-treated cloned placentae compared with ICSI-derived and NM control placentae. Together with the previous findings in mouse and bovine cloned placentae [17,18,19], our results suggest that cloned and TSA-treated cloned placentae, which maintain strong TIMP-2 expression at the end of gestation, are likely to exhibit decreased proteolytic activity and MMP-induced ECM degradation, potentially leading to placental dysfunction and enlargement.

The PBEF protein, in contrast, was downregulated in cloned, TSA-treated cloned and ICSI-derived placentae. PBEF appears to function at the proximal pathway for labor initiation [35], and is reportedly upregulated following distension of human amniotic epithelial cells [36]. We previous reported that the protein and mRNA expression levels of PBEF in normal mouse placentae gradually decreased from 11.5 to 18.5 dpc, but remained detectable throughout this period [24]. Similarly, a previous study found that PBEF is constitutively expressed in placentae and may play a role during normal pregnancy [37]. PBEF reportedly increases when fetal membranes are transiently distended [38], and it has been suggested to act as a growth regulator, facilitating the accommodation of placental tissue and preventing it from rupturing prematurely when advancing gestation increasingly distends the membranes [35]. In addition, PBEF was recently reported to have a local protective antiapoptotic effect in fetal membranes during placentation [38, 39]. Since distension of fetal membranes in vivo may increase apoptosis in the placenta [35], our present results and the previous reports collectively suggest a model wherein PBEF expression in the developing mouse placenta could protect against the fetal membrane distension-induced apoptosis of placental cells. Interestingly, PBEF expression was lower in cloned and ICSI-derived placentae than in NM control placentae. Placenta of survived ICSI fetuses have a normal level of TIMP-2 but a different level of PBEF compared with NM, which may cause a relatively mild placentomegaly in ICSI samples. These results indicate that insufficient levels of PBEF, which appears to be important for proper placental function, may be associated with the abnormal placental development and/or unsuccessful parturition of cloned and ICSI-derived animals [17, 40].

After cloning, the transferred nucleus must undergo epigenetic reprogramming in order to support successful development. Previous studies have shown that epigenetic reprogramming defects often occur in cloned embryos, as reflected by aberrant gene expression [15, 39,40] and abnormal DNA methylation patterns [14, 41,42]. The *sall3* locus, for example, has been shown to be an epigenetic hotspot for the aberrant DNA methylation associated with placentomegaly of cloned mice [43]. Thus, altered gene expression in the placenta appears to be at least partly due to abnormalities in DNA methylation. To examine whether the protein expression changes identified in the present work might reflect epigenetic programming defects, we investigated DNA methylation and histone acetylation in the promoter regions of *TIMP-2* and *PBEF* genes. No significant difference was observed in the methylation status of *TIMP-2* or *PBEF* among NM control, ICSI-derived, cloned and TSA-treated cloned placentae, indicating that the *TIMP-2* and *PBEF* genes are correctly methylated in the tested system and the methylation status may not appear to affect the mRNA expression of *TIMP-2* and *PBEF* in the placenta. In contrast, H3-K9 and -K14 acetylations of *TIMP-2* and *PBEF* analyzed by ChIP were up- and downregulated, respectively, in cloned placentae, in accordance with their observed mRNA and protein expression levels. Thus, cloned placentae must have suffered from aberrant reprogramming of histone changes in these (and potentially other) developmentally important genes, causing unusual expression of their proteins. These changes could give rise to the placental abnormalities seen in cloned mouse placentae, including enlargement and/or lack of proper placental function. Effect of TSA treatment in reconstructed embryos could be transient during embryo development. During early development, nuclei of reconstructed embryos treated with TSA could change acetylation status on H3K9 or globally on histone residues, and later, the modified epigenetic status could be erased or changed. Finally fetuses that have less severe modifications or proper changes in epigenetics could survive at term.

In sum, our findings indicate that TIMP-2 and PBEF are involved in abnormal hypertrophic placental development in the mouse. In cloned placentae, the failure of histone modification reprogramming in these (and potentially other) developmentally important genes appears to trigger the abnormal expression of their protein products. This is the first report to demonstrate that aberrant TIMP-2 and PBEF protein expression in the placentae of cloned mice is closely related to altered epigenetic modification of placental development-related genes.
